# Microcredit and willingness to pay for environmental quality: Evidence from a randomized-controlled trial of finance for sanitation in rural Cambodia^☆^

**DOI:** 10.1016/j.jeem.2016.11.004

**Published:** 2017-11

**Authors:** Ariel Ben Yishay, Andrew Fraker, Raymond Guiteras, Giordano Palloni, Neil Buddy Shah, Stuart Shirrell, Paul Wang

**Affiliations:** a William and Mary, USA; b IDinsight, USA; c North Carolina State University, USA; d IFPRI, USA; e Yale Law School, USA

**Keywords:** Sanitation, Microcredit, Becker-DeGroot-Marschak, Randomization inference, Decision-focused evaluation

## Abstract

Low willingness to pay (WTP) for environmental quality in developing countries is a key research question in environmental economics. One explanation is that missing credit markets may suppress WTP for environmental improvements that require large up-front investments. We test the impact of microloans on WTP for hygienic latrines via a randomized controlled trial in 30 villages in rural Cambodia. We find that microcredit dramatically raises WTP for improved latrines, with 60% of households in the Financing arm willing to purchase at an unsubsidized price, relative to 25% in the Non-financing arm. Effects on latrine installation are positive but muted by several factors, including a negative peer effect: randomly induced purchases by neighbors reduce a household's probability of installing its own latrine. On methodological grounds, this paper shows that a “decision-focused evaluation” can be integrated into academic analysis to provide insight into questions of general interest.

## Highlights

• We experimentally test the impact of microloans for latrines in Cambodia.
• Offering microloans dramatically raises willingness to pay.
• Installation rates of purchased latrines are low (35–40%) even after 18–24 months.
• Neighbors' purchases reduce a household's own latrine installation.
• We provide a model for addressing questions of academic interest from a “decision-focused evaluation”.

## Introduction

Environmental conditions are typically worse in poor countries along key dimensions such as clean air and clean water (WHO, 2014b, WHO, 2014a, WHO, 2015), and the burden of environmental disease similarly falls disproportionally on poor countries (Prüss-Üstün and Corvalán, 2006). However, willingness to pay (WTP) for environmental quality is typically low in developing countries, and understanding the reasons why is a key research question at the intersection of environmental and development economics (Greenstone and Jack, 2015).

Poor sanitation in developing countries is an important example of this general phenomenon. Globally, 2.5 billion people live without access to improved sanitation, with one billion of these people practicing open defecation (WHO, 2014). Inadequate sanitation is believed to cause 280,000 deaths per year (Prüss-Ustün et al., 2014), contribute to serious health problems such as chronic diarrhea and tropical enteropathy (Dangour et al., 2013, Lin et al., 2013), and may diminish human capital through impacts on stunting and cognitive capacity (Spears, 2012, Spears, 2013). Although simple, relatively affordable solutions such as low-cost pour-flush latrines exist and major policy initiatives have promoted their adoption, growth in latrine coverage and reduction in open defecation have been slow in many parts of the developing world.

Several explanations for this puzzle have been proposed, including lack of information on health benefits, peer effects and social influence, supply-side failures, and difficulties in coordinating in the face of externalities and complementarities (Pattanayak, 2009, Pattanayak et al., 2009, Trémolet, 2011, Perez et al., 2012, Gertler et al., 2015, Guiteras et al., 2015b). In this paper, we focus on one aspect of a household's decision to purchase and install a latrine: latrines require a large up-front investment whose benefits are realized over time. This is a common characteristic of many actions required to improve environmental conditions and health, and could limit investment for any of several reasons (e.g., lack of consumer credit, high discount rates, or present bias) and suggests that interventions to fill missing capital markets may increase investment and improve welfare. However, there is little evidence on the effectiveness of credit-based interventions at affecting WTP for environmental quality (Greenstone and Jack, 2015), and recent research showing limited impacts of micro-credit interventions on income and welfare has led to increased skepticism of the value of micro-finance (Banerjee, 2013, Banerjee and Karlan, 2015).

This paper reports the results of what is, to our knowledge, the first randomized-controlled trial of the effect of micro-loans on WTP for improved sanitation. In a representative sample of 30 villages in rural Cambodia, our NGO partner conducted group information sessions and sales meetings to market a low-cost, hygienic latrine. In 15 randomly selected villages, households were offered the option of financing their purchase with a loan from a local micro-finance organization. In the remaining 15 villages, sales were made on a standard lump-sum, cash-on-delivery basis. To maximize the precision of our estimates and broaden the set of research questions we are able to address, we used the Becker-DeGroot-Marschak (BDM) mechanism to obtain precise measures of WTP for each household (Becker et al., 1964).

We find that the offer of a micro-loan dramatically increases WTP. Mean WTP in the Financing arm is $51.8, as compared to $29.9 in the Non-financing arm. This large increase in WTP occurs across quantiles and among both the poorest and relatively less-poor households.^1^ At the approximate break-even price of 40, only 25% of households agree to purchase the latrine without financing, but more than 60% of households offered financing are willing to pay this full, unsubsidized price. Because NGOs and social enterprises face large village-level fixed costs in marketing and delivery, these increases in WTP can increase the cost-effectiveness of interventions, even net of the cost of providing financing, by amortizing these fixed costs over a greater number of sales.

The impact of finance on latrine coverage, while positive, is not as large as the effect on WTP. Latrine installation rates 1.5–2 years after the sale were low in both arms, muting the effect of finance. The primary barrier to installation was the high cost of households' desired latrine superstructure (walls and roof), for which financing was not offered. A secondary barrier, which we identify using the quasi-random variation in latrine purchases produced by the BDM mechanism, is a negative social spillover: exogenous increases in neighbors' purchases lead to lower installation rates and, ultimately, lower latrine coverage. This is consistent with several potential mechanisms: shared use of the latrines creating negative strategic complementarities in latrine installation – i.e. the private return to installation is decreasing in neighbors' latrine ownership - as well as with strategic substitutability in health investments.

This paper provides a model for the integration of “decision-focused evaluations” with academic analysis of general-interest questions. A decision-focused evaluation is one demanded by an implementer, designed to rapidly inform a specific policy or programmatic decision, and carried out in the specific context of interest and within in the implementer's usual operating and decision-making structures (Shah et al., 2015).^2^ The randomized evaluation in this paper was designed to inform our implementation partner's decision of whether it should scale up microfinance loans for latrines in rural Cambodia. The evaluation took 3.5 months to complete from inception to reporting results, cost less than 60,000 USD, and provided a clear, actionable recommendation to the implementer. Given the decision-focus of this evaluation, the survey instruments used were designed to minimize the time and cost of the evaluation, focusing only on collecting data that would be necessary for guiding iDE's decision. This focus limits our ability to investigate mechanisms behind the effects we observe, particularly our ability to distinguish credit constraints from present bias or high discount rates as the source of the effectiveness of the financing offer. At the same time, we are still able to provide rigorous evidence on an important question of broad interest.

The paper is organized as follows: in Section 2, we describe our setting and experimental design, including our study sample, intervention, and data collection. In Section 3, we show the dramatic effect of finance on demand. In Section 4, we show that the effect on coverage was less and explore reasons for this gap. In Section 5, we discuss the results, including the implications for cost-effectiveness, and conclude.

## Experimental setting and design

We conducted our experiment in rural Kampong Thom province, Cambodia, a region with generally low access to improved sanitation: as of 2012, 31% of rural residents had access to a hygienic latrine. At baseline, 71% of respondents in our sample primarily defecate in the open, with 90% of children under 5 in these households doing so.^3^ These children experience diarrhea regularly, with 39% of children in sample households having diarrhea in the 7 days preceding the survey.

Formal financing is not uncommon, with 41% of households having held a loan from a bank or microfinance institutions in the preceding year. Consumer loans from formal sources are very rare; most loans are for productive assets only. Informal credit is more prevalent (61% of households), but average loan holdings are relatively small.

We partnered with two institutions to implement the sanitation marketing and the microfinance loans. iDE Cambodia (iDE) has conducted sanitation marketing and training of sanitation suppliers in rural Cambodia since 2007 and currently is active in sanitation in eight provinces across Cambodia. Microloans were provided by VisionFund Cambodia (VFC), a microfinance institution established in 1994 that has served more than 140,000 clients with a total loan portfolio over US $37M.^4^ iDE had worked in Kampong Thom province, but not in any of the sample villages, while VisionFund was active in all.

As a demand-driven, decision-focused evaluation, the study design was tailored to the implementers' budgetary, timeline, and operational constraints and the intervention mimicked the implementers' standard procedures as closely as possible. However, all data were collected by employees of IDinsight, independently of the implementers.

### Sample frame and randomization

The study sample consists of 1,500 households from 30 villages in Kampong Thom province, selected using a multi-stage random sampling process. The initial village-level sample frame consisted of all 786 villages in Kampong Thom. Villages were then excluded from the sample frame on the basis of two criteria. First, to facilitate the selection of a representative sample, we dropped villages above the 95th percentile and below the 5th percentile with respect to population and share of households classified as “IDPoor.”^5^ Second, we excluded villages targeted by the Asian Development Bank's concurrent Rural Water Supply and Sanitation Sector Project, which provided heavily subsidized or free sanitation facilities to 75% or more of households. From the remaining villages, samples of 30 were randomly selected without replacement and with a probability of selection weighted by village size (number of households). At the end of each draw, we calculated the mean size, poverty rate and latrine coverage rate (measured at the district level) for the sample and compared these to the averages for Kampong Thom. If the sample differed significantly from the population (Kampong Thom as a whole), we discarded that sample. We repeated this procedure until it produced 100 qualified samples. Of these 100 qualified samples, one was randomly selected as binding. From this sample of 30 villages, 15 were randomly assigned to receive the Financing treatment.

In each village, a census was taken to obtain the names of the head of household and spouse and IDPoor status of the household, and to identify whether each household owned a latrine. Fifty households (average village size was 194 households) in each village were invited to participate in a group information session and sales meeting. These 50 households were randomly selected from the village population without a latrine, after stratifying on IDPoor status so that 30% of the selected households (i.e., 15 in each village) were classified as IDPoor. In four villages, fewer than 50 households did not have a latrine. In these four villages, all non-latrine-owning households were invited, yielding a total sample size of 1,383 households. On average, 76% of invited households attended the sales meeting. The household could be represented by any household member older than 18 with the authority to make large purchase decisions for that household. Field staff then followed up with non-attending households to conduct the same information and sales session, typically within one day of the initial session in the village. Ultimately, 1,380 of 1,383 invited households (99.8%) participated. Households were not compensated for attending the sales meeting, nor for any aspect of participation.

As described below, the intervention involved randomization of latrine prices at the household level. This randomization was unstratified and thus produced village-level variation in the average price draw.

### Group information session

The core of the intervention was the group information session and sales meeting.^6^ Invited households gathered in a common location (e.g. school, village pagoda, village chief's house). Sales staff led a 45–60 minute interactive session emphasizing the health and convenience benefits of having a latrine and its status as an aspirational good. The latrine offered for sale included three concrete rings, each 80 cm in diameter, a concrete pan, a concrete slab with a porcelain bowl that fits into the pan, and a PVC pipe that connects the three rings to the concrete pan. The approximate cost of this set of parts was 160,000 KHR (USD 40).^7^ Participants were not given information on the cost. The latrine was marketed as being easy to self-install, requiring approximately one day's labor to dig a 1.5×1 m cylindrical pit to house the three concrete rings and a separate mound to keep the basin. No additional material was required to install the latrine after delivery other than a shovel and water to mix the mortar. The sale did not include installation or a superstructure.^8^

### Financing treatment and sale

At the end of the information session, attendees were offered an opportunity to purchase a latrine. The sales offer was made using the Becker-DeGroot-Marschak (BDM) mechanism (Becker et al., 1964). In the Non-Financing (cash-on-delivery) arm, households were commonly anchored on an initial price of 300,000 KHR (USD 75), with prices subsequently reduced in 10,000 KHR (USD 2.5) increments until the participant was willing to accept that price. Prices were then increased in smaller increments (2,000 KHR) until the participant was no longer willing to pay. This mechanism identified the maximum WTP, or “bid”, for the latrine. The enumerator then allowed the household to choose from a set of sealed envelopes marked only with the participant's ID, each containing a randomly chosen price. The distribution of prices was 80,000 KHR, 120,000 KHR, 160,000 KHR, and 200,000 KHR (USD 20, 30, 40 and 50) with probability weights 1/4, 1/4, 1/4, 1/4 in the non-financing arm and 1/20, 9/20, 1/4, 1/4 in the financing arm, respectively.^9^^,^^10^ If the price inside the chosen envelope was less than or equal to the subject's bid, then the subject purchased the latrine at the randomly determined price. If the price in the envelope was greater than the subject's bid, then the subject could not purchase the latrine. The subject was not allowed to change her bid after the price was revealed. For expected utility maximizers, the subject's best strategy is to bid her maximum WTP.^11^ The bids were given and the price revealed in private. All subjects also took part in a practice round in which participants bid for a token item (a box of cookies worth approximately KHR 2,000) to ensure they understood the procedure.

The study of credit constraints involved two treatment arms: Lump Sum (control) and Financing (treatment), which were randomly assigned at the village level (as discussed above). In Lump Sum villages, the household was required to pay the full agreed price upon delivery, which would occur within 10 days. In Financing villages, the household was offered a loan from VFC, which could be repaid over a term of up to 12 months.^12^ The loans were group liability with monthly interest rates of approximately 2.8%.^13^ The sales mechanism was the same in Lump Sum and Financing villages, with two exceptions. First, in Financing villages, the loan option was explained before the bidding so that subjects could take this information into account when deciding their maximum WTP. Second, as indicated above, in Lump Sum villages, bids were made in terms of the full payment amount, while in Financing villages bids were made in terms of the monthly installment payments.^14^ Finally, in Financing villages, typically within 24 h of the group sales meeting, winning households met with a VFC underwriter, who used a basic battery of questions on the customer's age, income and assets to determine his eligibility for a loan. The underwriter then determined whether or not to extend the loan.^15^

To avoid non-random selection into attendance at the sales meeting, the treatment status of villages was not announced until the meeting itself. In fact, in Finance villages, no mention was made of the possibility of finance until the sales meeting, and in Lump Sum villages, the possibility of a loan was not mentioned at all. Households who were invited to the group meeting but did not attend were visited by enumerators within one day and offered an opportunity to participate at home. If they agreed, the information session and sales exercise were conducted in similar fashion to the group meeting.^16^

### Data collection

We collected data using four instruments. First, we administered a census to all households in each village, obtaining the name of the head of household and spouse, whether the household owned a latrine, and whether the household was classified as IDPoor. Second, a baseline survey was conducted with all invited, consenting households. The survey covered latrine type and conditions, defecation practices, knowledge of latrine components and costs, household demographics, informal borrowing of rice or money from other people, housing quality, income sources, land holdings and agricultural production.

Third, we also obtained data from the sales exercise. For household h in village v, BDM provides data on willingness to pay (${\mathrm{WTP}}_{\mathrm{hv}}$), the price offer (${\mathrm{Draw}}_{\mathrm{hv}}$), and whether the household won the latrine (${\mathrm{Won}}_{\mathrm{hv}}=1\left\{{\mathrm{WTP}}_{\mathrm{hv}}\geq {\mathrm{Draw}}_{\mathrm{hv}}\right\}$). We also record whether the household actually purchased the latrine (${\mathrm{Bought}}_{\mathrm{hv}}$). If the household won the latrine but reneged, that is recorded as a refusal: ${\mathrm{Refuse}}_{\mathrm{hv}}=\left({\mathrm{Won}}_{\mathrm{hv}}=1\right)⋂\left({\mathrm{Bought}}_{\mathrm{hv}}=0\right)$. For households that purchase the latrine, the price paid is the price offer, ${\mathrm{Draw}}_{\mathrm{hv}}$. The price paid is not defined for households that do not purchase. We also document whether households that lost attempted to bargain for the latrine and whether ex-post they wish they had bid more. For Financing villages, we convert to the declining balance sequence of payments to NPV using the interest rate of 2.8% per month as a proxy for VFC's cost of funds.

Finally, we conducted a follow-up survey to assess installation and use of the latrine via both self-reports and direct observation. The survey revisited all participating households approximately 18–24 months after the initial sales offers. Enumerators observed whether latrines (from any source) were installed, as well as conditions of installation, presence of a superstructure, and indicators of regular use and maintenance.

### Summary statistics

Table 1 presents baseline summary statistics and measures of balance for the sample. Column 1 presents means for the entire sample with standard deviations shown below in parentheses. Columns 2 and 3 present the means and standard deviations for Non-financing and Financing households, again with standard deviations shown in parentheses below each mean. Column 4 presents the difference in means between the Non-financing and Financing groups with standard errors presented in brackets below each difference. Finally, Column 5 presents the normalized difference $\left({\overset{¯}{X}}_{1}-{\overset{¯}{X}}_{0}\right)/\sqrt{\left(s_{0}^{2}+s_{1}^{2}\right)}$ between the two means (Imbens and Wooldridge, 2009).

**Table 1:** Summary statistics and balance.

	All households	Non financing	Financing	Diff.	Norm. diff.
	(1)	(2)	(3)	(4)	(5)
Female respondent	0.811	0.806	0.816	0.010	0.018
	(0.391)	(0.396)	(0.388)	[0.027]	
Household Size	4.382	4.294	4.467	0.173	0.069
	(1.781)	(1.783)	(1.776)	[0.170]	
Number of women in household	2.243	2.178	2.305	0.126	0.079
	(1.139)	(1.125)	(1.149)	[0.090]	
Any children under age five	0.453	0.473	0.433	−0.040	−0.056
	(0.498)	(0.500)	(0.496)	[0.028]	
Any children under age two	0.252	0.253	0.251	−0.002	−0.003
	(0.434)	(0.435)	(0.434)	[0.028]	
# members who earn income	1.693	1.781	1.607	−0.175	−0.106
	(1.165)	(1.229)	(1.093)	[0.130]	
Total monthly household income (USD)	122.815	134.800	111.029	−23.772	−0.039
	(431.070)	(560.843)	(243.357)	[37.409]	
Household owns livestock	0.825	0.826	0.825	−0.001	−0.002
	(0.380)	(0.380)	(0.380)	[0.040]	
Household grows crops	0.833	0.865	0.806	−0.058	−0.111
	(0.373)	(0.342)	(0.396)	[0.054]	
Any formal loan in past year	0.414	0.405	0.423	0.018	0.026
	(0.493)	(0.491)	(0.494)	[0.051]	
Any informal loan in past year	0.655	0.643	0.667	0.024	0.036
	(0.475)	(0.480)	(0.472)	[0.032]	
Any current formal savings	0.017	0.019	0.015	−0.004	−0.021
	(0.131)	(0.137)	(0.123)	[0.009]	
Any current informal savings	0.841	0.795	0.886	0.091	0.178
	(0.366)	(0.404)	(0.318)	[0.069]	
ID poor household	0.275	0.273	0.277	0.004	0.006
	(0.447)	(0.446)	(0.448)	[0.031]	
Likelihood<2 USD a day	25.922	24.833	26.970	2.138	0.070
	(21.698)	(21.373)	(21.971)	[1.806]	
Primarily defecate in the open	0.703	0.688	0.718	0.030	0.046
	(0.457)	(0.464)	(0.450)	[0.074]	
# of diarrhoeal episodes	0.232	0.274	0.201	−0.072⁎⁎⁎	−0.180
	(0.285)	(0.296)	(0.273)	[0.020]	
Children<=5 defecate in the open	0.904	0.894	0.914	0.021	0.050
	(0.295)	(0.309)	(0.280)	[0.035]	
Children<=5 # of diarrhoeal episodes	0.390	0.472	0.323	−0.149⁎⁎⁎	−0.229
	(0.465)	(0.476)	(0.446)	[0.039]	
Has considered latrine purchase	0.946	0.942	0.950	0.008	0.025
	(0.226)	(0.234)	(0.218)	[0.014]	
Group sales meeting	0.769	0.787	0.753	−0.034	−0.057
	(0.422)	(0.410)	(0.431)	[0.037]	
Latrine offer price (USD)	35.764	34.734	36.752	2.018⁎⁎⁎	0.142
	(10.062)	(11.012)	(8.956)	[0.617]	

*Notes:* Table displays summary statistics for the whole sample (Column 1) and by treatment arm (Columns 2 and 3). Column 4 displays the difference between the mean in the Non-financing and Financing arms while Column 5 displays the normalized difference between the two means $\left({\overset{¯}{X}}_{1}-{\overset{¯}{X}}_{0}\right)/\sqrt{\left(s_{0}^{2}+s_{1}^{2}\right)}$. Standard deviations appear in parentheses while standard errors appear in brackets. ^⁎⁎^ p≤0.05, ^⁎^ p≤0.10. Child age cutoffs are inclusive of the cutoff age. The number of individuals who contribute income and total household income include non-resident members. The likelihood that the household lives on less than $2 per day is defined using the 2011 Progress out of Poverty Index (PPI). For variables in the PPI that were not included in the baseline survey we impute the mean value from the 2008 census in Cambodia for rural households in Kampong Thom province.

⁎⁎⁎ p≤0.01.

81% of respondents are female, and nearly 50% live in a household with a child five years old or younger (25% live in household with child two years old or younger). By design, just under 30% of households are IDPoor, and mean household monthly income is just over $120. Many households had been exposed to microfinance prior to the study: 41% had taken out a loan from a formal source in the previous year.

Open defecation is extremely common among sample households: 70% of all individuals and 90% of children under the age of five primarily defecated in the open over the fifteen days preceding the survey. Despite this, households are clearly familiar with sanitation options: nearly 95% had previously considered purchasing a latrine in the past.

While the Financing and Non-financing groups appear generally well balanced in terms of baseline characteristics, there are a few significant differences. Episodes of diarrhea over the week preceding the survey are significantly lower in the Financing group relative to the control group, both among all individuals and among children age five and younger. The BDM latrine offer price is, on average, $2 higher for Financing households than non financing households. To the extent that these baseline characteristics are predictive of successful latrine purchase through the BDM procedure, we would expect them to bias our results against finding any significant impact of Financing; that is, higher latrine offer prices should decrease latrine purchases in the Financing group relative to the control group. Similarly, the lower frequency of diarrhoeal episodes in Financing households may suggest they have smaller expected health returns to improved sanitation. Section 3.4 explores whether our main results are sensitive to these baseline differences.

## Effect of finance on demand

### Main results

**Fig. 1:**
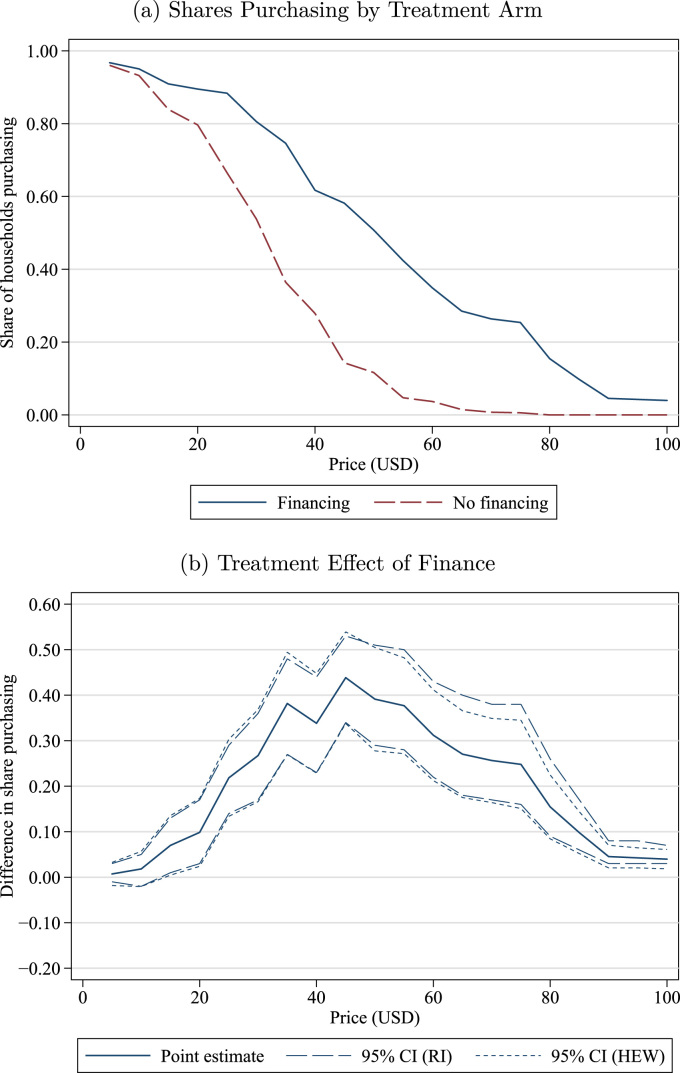
Levels of demand and effect of finance. *Notes:* The top panels shows the shares of households with willingness to pay greater than or equal to each price, by Financing and Non-Financing treatment. The bottom panel shows the treatment effect of finance, i.e. the estimated difference between the Financing and Non-financing treatments in the share of households with willingness to pay greater than or equal to each price, with 95% confidence intervals. This difference is estimated at $5, 10,…, 100. The RI confidence interval (long dashes) is computed by randomization inference, while the HEW confidence interval (short dashes) is the usual heteroscedasticity-robust standard error. Observations are at the village level, with each village weighted by the number of participating households.

We are interested in the effect of finance on the (inverse) demand curve, i.e., $s\left({\mathrm{WTP}}_{\mathrm{hv}}\geq p\right)$, the share of households willing to purchase at a set of prices $p_{L},\ldots ,p_{H}$. Fig. 1a plots $s\left({\mathrm{WTP}}_{\mathrm{hv}}\geq p\right)$ at each price p={5,10,…,100} separately by treatment group (Non-Financing and Financing). Households in both groups purchased latrines at relatively high rates (>80%) when the price is below 20 USD. Demand is quite elastic over prices in the [20,40] range (or 50–100% of market prices), especially in the Non-Financing group where only 27.9% of households would purchase the latrine at 40 USD.

In Fig. 1b, we plot the estimated treatment effect of finance, with 95% confidence intervals constructed using both randomization inference and standard regression methods. (We discuss randomization inference in Section 3.2 and in Aglasan et al., 2016) By randomization, this estimated treatment effect is the observed difference between the two demand curves in Fig. 1a. We weight each household equally but the effects are similar if we weight villages equally. We find large treatment effects that initially increase with price, reaching a maximum that exceeds 40 pp at 45 USD. Beyond this price, demand falls relatively quickly even in Financing villages, with treatment effects diminishing (although remaining greater than zero even at 100 USD). The lower bound for treatment effects exceeds 0 for all p≥20. At the approximate break-even or unsubsidized price of 40 USD, treatment effects are 33.8 pp (RI p-value=0.00), more than doubling the share of households purchasing latrines relative to the Non-Financing group.

One reason that financing may lead to higher purchase rates may be that households expect a substantial likelihood of defaulting. However, using administrative data from VisionFund, we track repayment rates for the ensuing year after the initial offer and find 100% repayment of the latrine loans.

### Estimation and inference

By virtue of randomization, obtaining point estimates for treatment effects is simple: at each price p, we can take the difference in shares purchasing, i.e.,

(1) β^(p)=sF(WTPhvF≥p)−sNF(WTPhvNF≥p).

Alternatively, we can use the estimated coefficient from a regression of $1\left\{{\mathrm{WTP}}_{\mathrm{hv}}\geq p\right\}$ on an indicator for whether household h's village was in the Financing treatment group, plus a constant.

Computing p-values and confidence intervals is not as straightforward. Because the treatment was randomized at the village level, and determinants of demand are likely correlated within village, inference must be made robust to clustering within village. However, because we have only 30 villages, the customary cluster-robust regression standard errors may be unreliable (Cameron et al., 2008).

Instead, we employ randomization inference both to obtain p-values and to construct confidence intervals by inverting the relationship between effect and p-value. While randomization inference for p-values has grown in popularity in recent years,^17^ accurate confidence intervals are important for policymakers because policy decisions usually depend on the magnitude of an effect, not just whether an effect exists, and use of randomization inference to construct confidence intervals is rare in economics.^18^ The intuition for randomization inference dates back to Fisher (1935): the researcher specifies a sharp null hypothesis, imposes this null hypothesis on the data, generates the distribution of the test statistic of interest under this null over all or many combinations of treatment assignments, and then compares the actual, observed value of the test statistic to its generated distribution to estimate how extreme the observed value is when the null is true (Rosenbaum, 2010, Imbens and Rubin, 2012). This procedure provides p-values directly when the null hypothesis is one of no effect. To obtain confidence intervals of level α, we test a series of null hypotheses (in our application, effects of −0.40,−0.39,…,+0.80) and construct the confidence interval as all values that are not rejected at the α level. We provide an extended discussion and formal exposition in a companion paper (Aglasan et al., 2016), and our code is available to interested researchers upon request.

For comparison, we also present “analytical” CIs computed using Huber-Eicker-White (HEW) heteroscedasticity-robust standard errors. We collapse the household-level data to the village-level share of households purchasing at price p, so, with one observation per village, clustering is not needed. However, this creates the possibility of small-sample bias in the HEW standard errors, so, following Angrist and Pischke (2009), we take the maximum of i.i.d, HEW, ${\mathrm{HC}}_{2},{\mathrm{HC}}_{3}$, where ${\mathrm{HC}}_{2}$ and ${\mathrm{HC}}_{3}$ are finite-sample corrections to HEW standard errors. As shown in Fig. 1b, the randomization inference confidence intervals are only slightly wider than the “analytical” CIs.

### Subgroup analysis

**Fig. 2:**
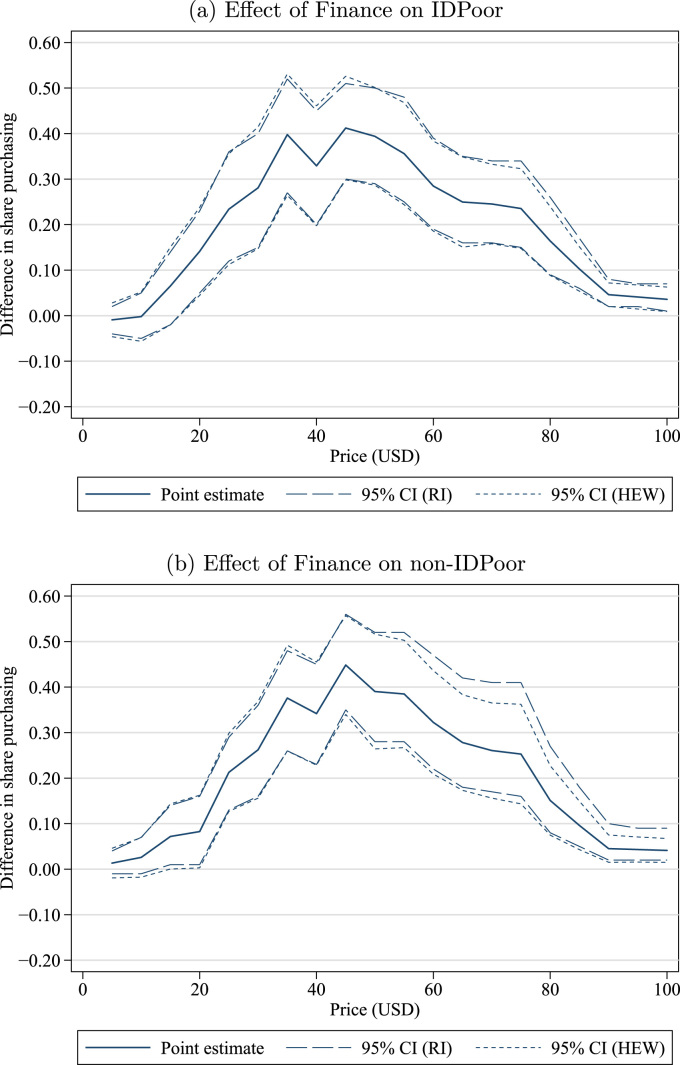
Effect of finance IDPoor vs. non-IDPoor. *Notes*: These figures show the estimated treatment effect of finance on IDPoor households (top panel) and non-IDPoor households (bottom panel). Each figure shows the estimated difference between the Financing and Non-financing treatments in the share of households with willingness to pay greater than or equal to each price, with 95% confidence intervals. This difference is estimated at $ 5, 10,…, 100. The RI confidence interval (long dashes) is computed by randomization inference, while the HEW confidence interval (short dashes) is the usual heteroscedasticity-robust standard error. Observations are at the village level, with each village weighted by the number of participating households.

One question of particular policy interest is whether the effect of finance differs by a household's initial poverty status.^19^ It is reasonable to think that poorer households' WTP will be relatively more responsive to access to financing. Poorer households have fewer financial assets (e.g., savings that can be drawn against to pay for a large lump-sum expense) and fewer physical assets (either for liquidation or to serve as collateral), and they may already be at sufficiently low levels of consumption that cutting consumption to finance an investment in a durable good carries a high utility cost. On the other hand, perhaps even with the availability of finance, poor households may find other consumption needs to be higher priorities, whereas relatively better-off households may find that the financing option makes purchasing the latrine more attractive. Fig. 2 presents estimated treatment effects by IDPoor subgroup, showing that WTP increased similarly in both subgroups. In fact, the demand curves in Non-Financing villages for poor and non-poor households are also remarkably similar. (See Fig. S1 in the Appendix A.) This is somewhat puzzling, since we expect sanitation to be a normal good. Our initial hypothesis was that IDPoor status may have been manipulated, or was a poor proxy for relative poverty within these communities. However, this does not appear to be the explanation: we constructed an alternative measure of household socio-economic status using asset measures from our baseline survey and found (a) that our measure is correlated with IDPoor status and (b) levels of demand and effects on demand were also generally similar when we used this alternative variable to classify households as rich or poor. One possible explanation is that the sample frame consisted of households without latrines at baseline, so the less-poor households are implicitly selected for having relatively low interest in sanitation in spite of their socio-economic status.

### Robustness checks

#### Cancelled orders and loan rejections

There were a small number of households who won a latrine during BDM but then cancelled their order upon latrine delivery. These “cancelling” households make up 2.5% of winners, but are disproportionately found in the Non-financing group: they represent 4.4% of winners in the Non-financing group and just 0.71% of winners the Financing group. One concern with respect to cancelling households is that their stated WTP in the BDM procedure may not represent their true WTP for the latrine. Given that these households end up electing to cancel the transaction at their stated WTP, it is possible that they overstated their true WTP. Similarly, 7.9% of winning households in the financing arm had their loan rejected by VFC, so their BDM bid likely overstated what they truly were willing and *able* to pay.

**Fig. 3:**
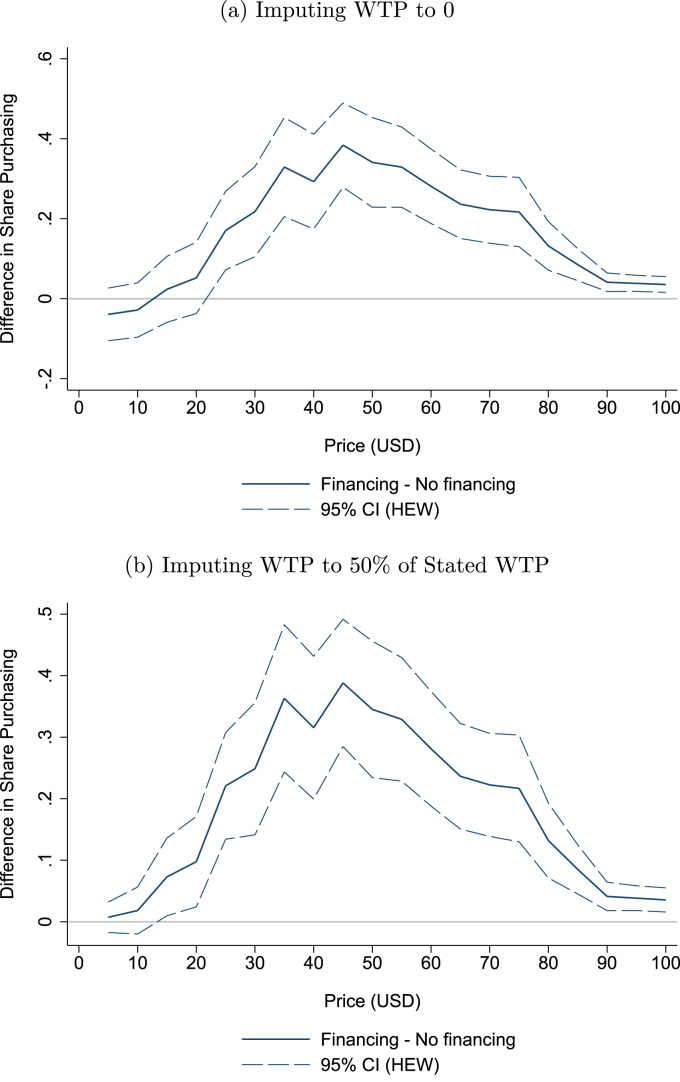
Different assumptions about true WTP households cancelling order or rejected for loan. *Notes:* Figures show the robustness of the main results to two different assumptions about the true willingness to pay (WTP) of households who either cancelled their purchase upon latrine delivery or were not approved for a loan by the lender. The top panel assumes true WTP is 0 for all cancelling or rejected household while the bottom panel assumes true WTP is 50% of stated WTP. Both panels show the estimated difference between the Financing and Non-financing treatments in the share of households with WTP greater than or equal to each price, with 95% confidence intervals. Confidence intervals are based on heteroskedasticity-robust standard errors from a village level regression weighted by the number of participating households. Confidence intervals based on 999 cluster-robust bootstrap repetitions are nearly identical.

To test whether our results are robust to these considerations, Fig. 3 re-estimates the differences in the share of households purchasing the latrines under different assumptions about the true WTP for households who end up cancelling their order and households who are not approved by VFC.

We begin by making the most extreme assumption possible: Fig. 3a assumes that all households who cancelled their purchase or who were rejected by VFC for a loan have a true WTP of $0. While the difference in the share of households purchasing the latrine at prices between $20 and $100 appears slightly smaller, it remains statistically significant and quite large in magnitude. At the market price of $40, the estimated difference in the share of households purchasing the latrine is still 30 pp when assuming true WTP is zero for these households.

Fig. 3b makes a less extreme assumption about true WTP for these households: that their true WTP is 50% of their stated WTP. Given that our results were largely unaffected when assuming true WTP was 0% of stated WTP, the estimated differences in the share of households purchasing the latrine at each price are unsurprisingly close to our main estimates in Fig. 1b. At the market price, the estimated treatment effect of Financing drops to 31.5 pp, just slightly lower than the 33.8 pp difference we find when using stated WTP.

**Fig. 4:**
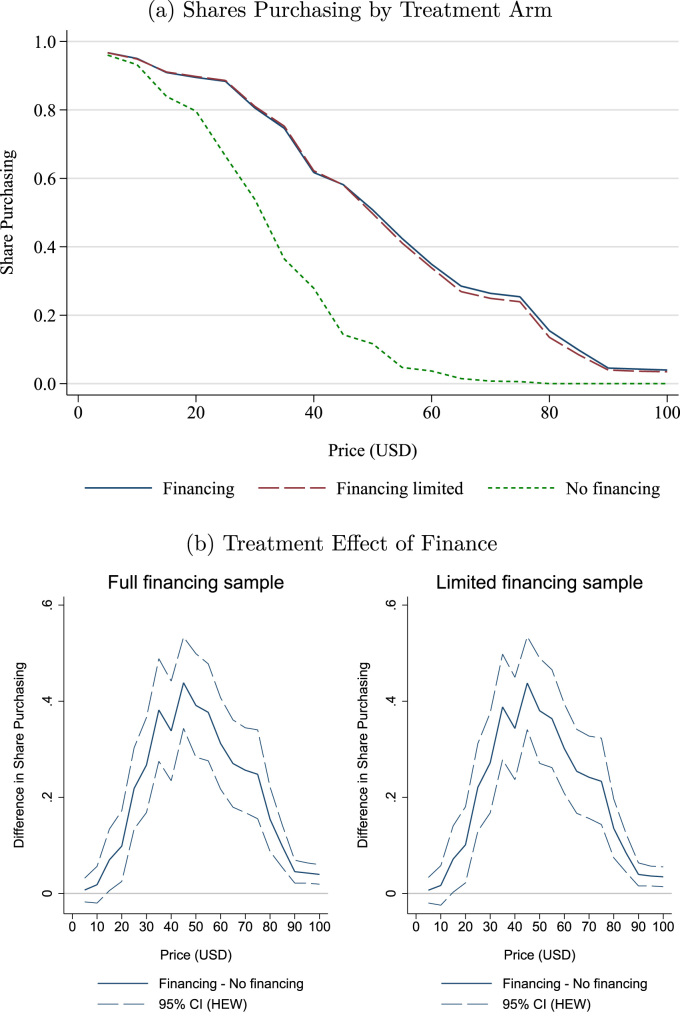
Dropping villages with high loan rejection rates. *Notes*: Figures show the robustness of the main results to dropping two villages in the Financing treatment group where loan rejection rates were high. The top panel displays the share of households with willingness to pay (WTP) greater than or equal to each price by treatment arm. Financing treatment shares are shown when including all villages (Financing) and when dropping the aforementioned villages (Financing limited). The bottom panel shows the estimated difference between the Financing and Non-financing treatments in the share of households with WTP greater than or equal to each price, with 95% confidence intervals when including (left) and not including (right) the high loan rejection rate villages. Confidence intervals are based on heteroskedasticity-robust standard errors from a village level regression weighted by the number of participating households. Confidence intervals based on 999 cluster-robust bootstrap repetitions are nearly identical.

The vast majority of loan rejections occurred in two villages where approximately 25% of households were rejected, as compared to less than 3% of households in the other 13 financing villages. Anecdotally, it seems VFC had experienced high rates of partial or full default during previous lending activities in these villages and was especially cautious about extending loans. It does not appear that WTP was unusually high or low in these two villages. As shown in Fig. 4, which displays our main results both when retaining the two high rejection rate villages and when dropping them from the sample, WTP in these two villages was not noticeably different from the other villages in the financing treatment.

#### Baseline controls

**Fig. 5:**
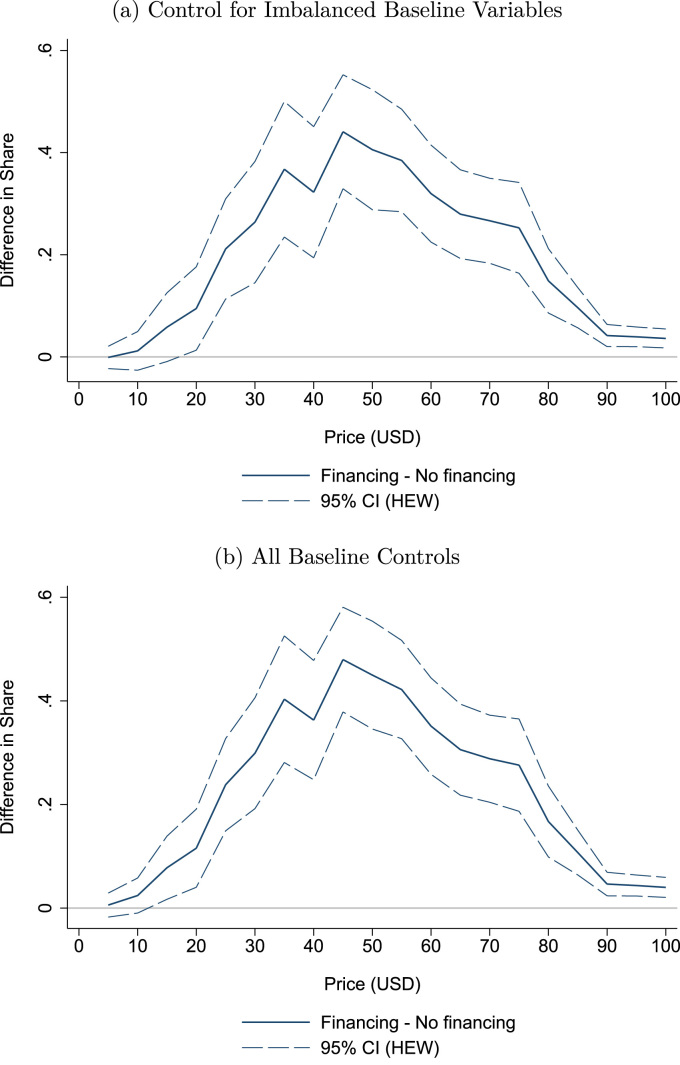
Including controls for unbalanced baseline characteristics. *Notes*: Figures show the robustness of the main results to controlling for baseline characteristics. Both panels present the coefficient and 95% confidence interval on the Financing dummy from an individual-level linear probability model where the dependent variable is an indicator for whether the household would purchase the latrine at each price. The top panel includes controls for variables from Table 1 that were imbalanced at baseline (BDM draw price dummy variables and the household average number of diarrhoeal episodes experienced over the week preceding the survey). The bottom panel controls for all baseline variables listed in Table 1. Households with missing values are coded to 0 and an indicator variable is included for each baseline covariate that is equal to 1 if the households had a missing value and 0 otherwise. Confidence intervals are based standard errors clustered at the village level. Confidence intervals based on 999 cluster-robust bootstrap repetitions are nearly identical.

A third potential concern with our main results is that they may be, in part, driven by the baseline differences shown in Table 1. To account for this possibility, Fig. 5 displays the estimated effect of financing on the share of households purchasing the sanitary latrine at each $5 increment between $0 and $100, controlling for covariates. In Fig. 5a, we control for the variables with significant differences in Table 1: BDM offer price indicators ($30,$40,$50) (with $20 being the omitted category), and the mean household number of diarrhoeal episodes experienced during the week prior to the survey.^20^ In Fig. 5b, we control for all baseline characteristics reported in Table 1. In both cases, including controls leads to only minor differences in the estimates, and the main result – economically important and statistically significant differences in demand between the financing and no financing groups at all prices between USD 20 and USD 100 – is unchanged.

## Effects on latrine installation

The previous section provides strong evidence that finance increases demand for sanitation. However, increasing initial purchases of latrines is necessary but not sufficient for improvements in environmental quality: toilets cannot reduce fecal loads in the environment unless they are installed and used. We conducted a followup survey 18–24 months after the initial sale to measure installation rates and collect objective indicators of latrine maintenance and use. At followup, we found that roughly 30–40% of households that had purchased a latrine during the sales exercise had installed it.^21^ Although this is comparable with rates of increased coverage from other sanitation interventions,^22^ it is somewhat surprising that only 30–40% of households that had purchased a latrine (as opposed to simply being encouraged to install one or given one for free) would have installed it nearly two years later. In this section, we first describe installation rates as a function of WTP and Financing treatment, then provide evidence of the cost of the superstructure as a barrier to installation, and finally explore the role of peer effects in depressing installation.^23^

### Finance, WTP, and installation rates

**Fig. 6:**
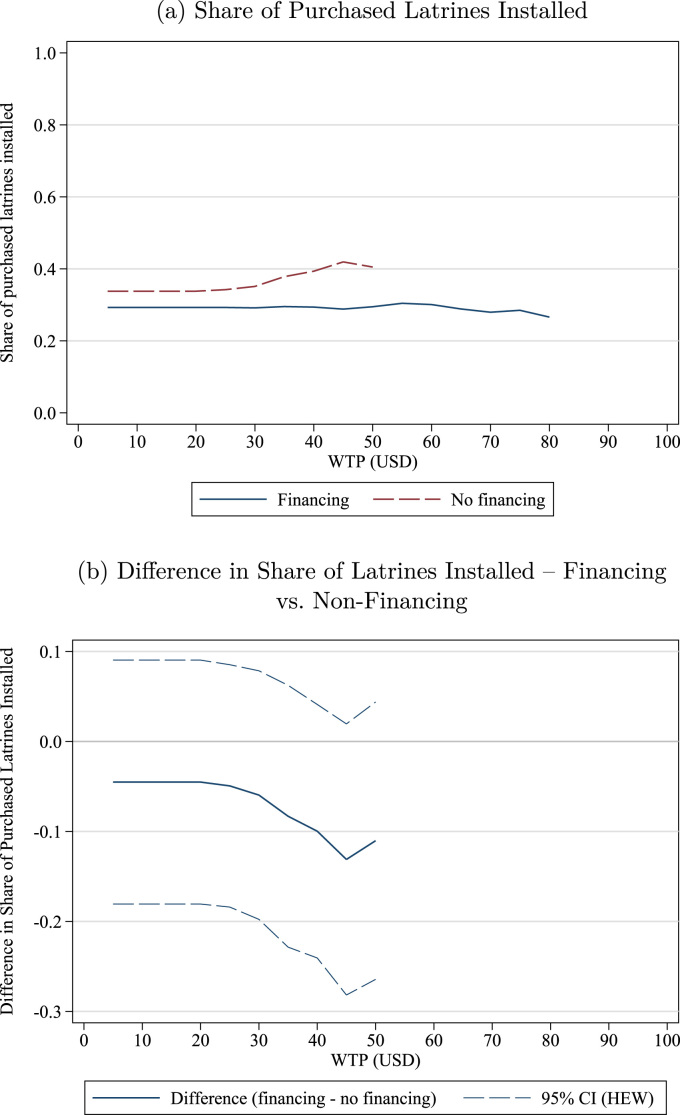
Latrine installation rates by WTP and financing treatment conditional on purchase. *Notes*: The top figure plots installation rates for households who purchase a latrine and have WTP greater than or equal to indicated price, separately by Financing and Non-financing treatment. The bottom figure plots the estimated difference in installation rates between households in the Financing and Non-financing treatments. The top figure is restricted to prices where 10% or more of the households in that treatment agreed to purchase a latrine (Non-financing: $50; Financing: $85), and the bottom figure is restricted to prices where 10% or more of the households in both treatments agreed to purchase a latrine ($50).

Fig. 6a plots, at each indicated price, the installation rate among all households that purchased the latrine and had WTP greater than or equal to that price. That is, the figure answers the question, “If an NGO offered latrines for sale at a given price, what share of latrines purchased at that price would be installed?” The figure is restricted to prices where 10% or more of the households purchased a latrine ($50 or less for Non-financing; $85 or less for Financing). There are three salient messages from the figure. First, installation rates are below 40% except among the Non-Financing households with WTP above $40. Second, installation rates at any given level of WTP are slightly higher among Non-Financing households than Financing, although these differences are not statistically significant (see Fig. 6b). Third, there is little evidence of screening effects: installation rates are roughly constant as a function of WTP, although there are slight differences in the right tails of the WTP distribution: installation rates are slightly increasing among Non-Financing households with relatively high WTP and slightly decreasing among Financing households with relatively high WTP.^24^

Fig. 6 shows installation rates conditional on purchase, but it is also useful to consider unconditional installation rates. That is, if latrines were offered for sale at a given price, what share of households would purchase and install a latrine? Because of the randomness of BDM, this unconditional installation rate has to be calculated indirectly. We would like to calculate

(2) s(Install)=s(Install|Purchase)s(Purchase).

However, the purchase decision, s(Purchase), has a random element since it depends in part on the BDM draw. To estimate the share that would purchase and install a latrine if the fixed price were p, we substitute s(Bid≥p) for *s* (Purchase). That is, we use the installation rate that would have occurred among participants if a fixed price of p had been offered (or if everyone bidding p or more had won).

**Fig. 7:**
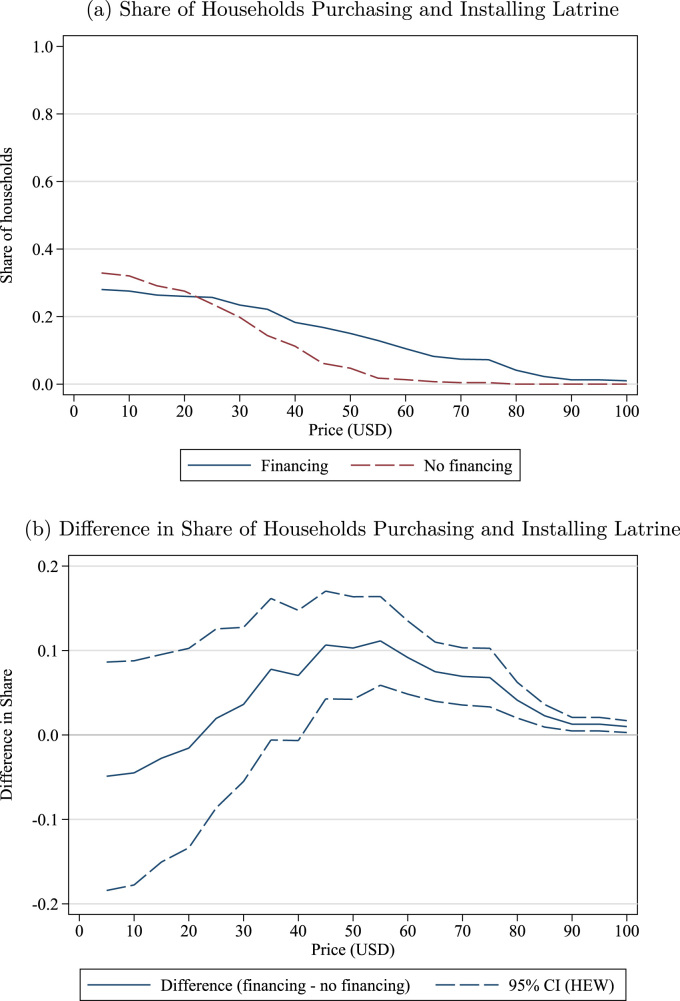
Latrine installation rates by price and financing treatment unconditional. *Notes*: The top figure plots the share of households that, at each price, would purchase a latrine at that price and subsequently install it, separately by Financing and Non-financing treatment. The bottom panel plots the estimated difference, with a 95% confidence interval, between households in the Financing and Non-financing treatments.

This synthetic installation rate is plotted in Fig. 7a as a function of price, separately for Financing and Non-Financing villages. Fig. 7a answers the question, “If an NGO offered latrines for sale at a given price, what share of households would purchase and install a latrine?” Note that the installation rate is now *higher* for Financing villages than Non-Financing at most prices, seemingly contradicting Fig. 6. However, the two figures are in fact consistent – as shown in Fig. 6, installation rates are higher in Non-Financing villages holding WTP constant, but WTP is much higher in Financing villages (see Fig. 1), so overall coverage rates are generally higher. This difference is statistically significant at all prices above $40, as shown in Fig. 7b.^25^

What explains these installation rates? First, the cost of the superstructure (walls, roof, and door) appears to have been an important barrier. Although is possible to build an inexpensive superstructure using locally gathered materials (bamboo or thatch) or tin (typically less than $10), households exhibit a strong stated preference towards much more elaborate, expensive concrete structures costing $200 or more.^26^ In informal discussions during the followup, households that had purchased a latrine but not yet installed it commonly stated that they intended to install the latrine eventually but had not yet saved enough for their desired superstructure. This suggests two approaches that might be effective at increasing installation rates: first, encouraging households to construct acceptable interim superstructures while they gather funds for the high-end superstructure they desire; second, by financing the superstructure in addition to the latrine itself. The former approach could be implemented by a subsidy, e.g., forgiving part of the loan if the latrine is installed. Cash-on-hand constraints do appear to matter to some extent: using the random variation in price paid generated by the BDM draw, we find that each $10 reduction in the price paid (holding WTP constant) by the household increases the probability of installation at followup by approximately 4% points. See Appendix D for the details of this analysis.

### Peer effects and installation

A second possible explanation for the low installation rate comes from a negative peer effect. Adoption of health technologies by nearby households has been shown to powerfully alter one's own beliefs about and propensity to adopt the technology (Godlonton and Thornton, 2012, Grant Miller and Mushfiq Mobarak, 2014). Moreover, peer effects may arise through varied mechanisms, some of which induce greater own adoption (learning, imitation) and others reduce it (sharing).^27^ The net effect of adoption by peers is thus ambiguous, and we seek to assess its sign and magnitude. Peer effects are typically difficult to identify, because observed correlation in behavior could be the result of a true, causal peer effect or simply homophily: peers tend to have similar tastes, so we might expect their behaviors to be similar even absent a true, causal effect (Manski, 1993). However, here we can use the randomization embodied in BDM to identify peer effects in installation. Because the price draw in BDM is random, there will be some random variation in latrine purchase rates in households’ peer groups. Furthermore, since BDM provides data on the household's WTP and the WTP of peer households, we can control for these and use only random variation in purchase rates conditional on own and peer WTP.

Ideally, one would collect detailed social network data to observe each household's relevant peer group precisely (BenYishay and Mushfiq Mobarak, 1964, Barrios et al., 2015). However, this was not feasible in this “decision-focused evaluation” because of time and budget constraints. Instead, we define each household's peer group as the other participating households in that household's village. Given the small size (on average, 250 households and 2–5 km diameter) and rural nature of the villages in our sample, it is plausible that the resulting village groups are not far from the true peer groups relevant for latrine ownership, installation, and use.

Our goal is to estimate the causal effect of latrine purchases by a household's peer group on that household's propensity to install a latrine. That is, we wish to estimate

(3) $$1\left\{{\mathrm{Install}}_{h,v}\right\}=\beta_{0}+\beta_{1}s\left({\mathrm{Buy}}_{∼h,v}\right)+\varepsilon_{h,v},$$

where $1\left\{{\mathrm{Install}}_{h,v}\right\}$ is an indicator for whether household h in village v has installed a latrine at the time of the followup survey, and $s\left({\mathrm{Buy}}_{∼h,v}\right)$ represents the share of other households (excluding household h) in village v who purchased a latrine. However, OLS estimation of Eq. (3) may produce biased estimates of the causal parameter of interest $\beta_{1}$. The most plausible source of bias would be if households' preference for improved sanitation tended to be correlated within village, so in villages where WTP was greater and, therefore, $s\left({\mathrm{Buy}}_{∼h,v}\right)$ tended to be higher, households were also more likely install a latrine they had purchased (i.e., $\varepsilon_{h,v}$ was higher on average).

To overcome this identification problem, we can use the randomness provided by the price draw in BDM. The simplest strategy would be to instrument for $s\left({\mathrm{Buy}}_{∼h,v}\right)$ with ${\overset{¯}{\mathrm{Draw}}}_{∼h,v}$, the average price draw among household h's peer group. We can increase power by using our knowledge of the distribution from which prices were drawn to define each household's chance of winning a latrine based on their bid. For example, for a household with a sufficiently low WTP for the latrine (i.e., below the minimum possible price), the realized price draw will not have any predictive power for whether the household purchased the latrine. By focusing on the randomly induced difference between actual latrine purchases and those predicted by the households' WTP, we use only the price variation that can affect latrine purchase. Households with high WTP will be expected to purchase the latrine under most potential price draws, while the opposite is true for households with low WTP. The effect of price draws are thus conditional on WTP.

We can thus construct the “unexpected” purchases among each household's peer group, defined as s˜(Buy∼h,v)=∑j≠h[(Buyj,v)−(Buy^j,v)], where Buy^j,v is household j's probability of purchasing given its WTP. We use the empirically observed distribution of price draws and the elicited WTP for each household to calculate their ex-ante probability of winning the latrine. As described in Section 2.3, latrine prices were drawn from a discrete distribution of $20, $30, $40, $50, with probabilities 1/4, 1/4, 1/4, 1/4 in the non-financing arm and 1/20, 9/20, 1/4, 1/4 in the financing arm, respectively.^28^ Therefore, any household with WTP<20 had a 0% chance to win the latrine, any household with 20≤WTP<30 had a 1/4 chance to win in the non-financing arm and a 1/20 chance to win in the financing arm, households with 30≤WTP<40 had a 1/2 chance to win, households with 40≤WTP<50 had a 3/4 chance to win, and those with 50≤WTP had a 100% chance to win. We can then calculate expected latrine purchase counts at the village level by summing these probabilities over all other participating households in the village.

Table 2 displays summary statistics for unexpected latrine purchases, $\overset{˜}{s}\left({\mathrm{Buy}}_{∼h,v}\right)$, as well as the observed latrine installation rates at endline. On average, households are exactly as likely to have won a latrine through BDM as their WTP and the ex-ante distribution of potential draw prices would predict (mean and median of unexpected latrine ownership is 0.00), and the range of observed values is −0.75 to 0.95. In words, a value of unexpected latrine purchase of −.75 implies that a household in the non-financing arm bid between [40 and 50 USD), but received a price draw of 50 and therefore did not win the latrine. A value of unexpected latrine purchase of.95 implies that a household in the financing arm bid between [20 and 30 USD) and drew a price of 20 USD, winning the latrine in spite of their relatively low WTP for the latrine. Higher values of unexpected latrine purchase are found for households that were especially lucky to win the latrine through BDM given their stated WTP.

**Table 2:** Unexpected latrine purchase and latrine installation summary statistics.

	mean	sd	min	p50	max
Unexpected latrine purchase	0.00	0.34	−0.75	0.00	0.95
Latrine installed	0.22	0.41	0.00	0.00	1.00

*Notes*: Estimates reflect full sample of 1,363 households. Unexpected latrine purchase is the difference between a household's actual latrine purchase and its expected purchase, calculated based on the empirically observed probability that its price draw falls below its stated WTP. Latrine installed is an indicator for whether the household had installed improved sanitation by the endline.

Although we can simply estimate the effects of peers’ unexpected purchases on latrine installation, we want to maximize precision by controlling for a household's own predictors of installation, including own latrine purchase. We do so by controlling for the randomly induced latrine purchase based on a household's own price draw and WTP. That is, we construct the own-household equivalents of our peer measures as Buy^h,v=Pr(Draw≤Bidh,v) and Buy˜h,v=Buyh,v−Buy^h,v. The latter term, ${\overset{˜}{\mathrm{Buy}}}_{h,v}$, represents the unexpected or random component of latrine ownership.

Using these variables, we estimate

(4) $$1\left\{{\mathrm{Install}}_{h,v}\right\}=\beta_{0}+\beta_{1}\overset{˜}{s}\left({\mathrm{Buy}}_{∼h,v}\right)+\beta_{2}{\overset{˜}{\mathrm{Buy}}}_{h,v}+\gamma \prime x_{h,v}+\varepsilon_{h,v},$$

by OLS, with results reported in Table 3. The covariates $x_{h,v}$ are an indicator for whether the village was a part of the Financing treatment, the household's WTP in USD, and the village mean WTP in USD excluding the household's own WTP. Rather than base inference on asymptotic cluster robust methods, we estimate p-values and confidence intervals for each parameter of interest through cluster robust bootstrap procedures: the Wild bootstrap (Cameron et al., 2008) in OLS specifications and the Score bootstrap (Kline and Santos, 2012) in IV specifications. We use cluster-robust bootstrap methods instead of randomization inference primarily because the literature on randomization inference for IV (e.g. Rosenbaum, 2002, Small et al., 2008) focuses on a binary instrument and extending the methods to multi-valued instruments, as in the case of the price draw, was beyond the scope of this paper. Since bootstrapping and randomization inference produced very similar results in our other analyses (purchase shares, mean WTP, WTP quantiles), we are comfortable using only the bootstrap for inference in our analysis of peer effects.

**Table 3:** Unexpected village latrine purchase and household installation.

	Installation Rate (OLS)	P-value
Village unexpected purchase	−0.910	0.039
	(−2.326, −0.046)	
Unexpected purchase	0.150	0.000
	(0.076, 0.224)	
Observations	1363	

*Notes*: Estimates reflect full sample of 1,363 households. Unexpected purchase is the difference between a household's actual latrine purchase and its expected purchase, calculated based on the empirically observed probability that its price draw falls below its stated WTP. Village unexpected purchase is the mean of unexpected purchase across a respondent's village, leaving out the respondent's own values. An indicator for whether respondent was in a financing village, the respondent's WTP (in USD), and the mean village WTP (in USD) excluding the respondent are included as controls. 95% confidence intervals shown in () beneath each estimate. P-values and 95% confidence intervals are based on 100,000 Wild bootstrap repetitions using Webb weights, clustering at the village level.

As expected, unexpected own purchase has a positive and significant effect on the likelihood that a household installed a latrine; the point estimate suggests nearly a 15% point increase in the probability of latrine installation. The fact that the estimate is not 1 is indicative of the relatively low installation rates in our sample. More interestingly, the coefficient on the village leave out mean unexpected latrine share is negative and statistically significant (p<.05). This suggests that, after removing the effect of WTP and conditional on own ownership, having more neighbors purchase a latrine actually reduces the likelihood that a household installs a latrine. The point estimate indicates that shifting a household to a village with an unexpected latrine share that is one standard deviation (.045% points, or ≈2.5 additional households in a typical village with 50 participating households) higher is expected to reduce the likelihood that household installs a latrine by 4.1% points, as compared to the mean installation rate of 35% among purchasers.

This reduced-form estimate identifies the effects of unexpected peer purchase on the likelihood that a household has installed a latrine at endline. One may naturally want to assess the effects of *any* peer purchase (rather than only unexpected purchases); that is, to estimate effects of $s\left({\mathrm{Buy}}_{∼h,v}\right)$ rather than $\overset{˜}{s}\left({\mathrm{Buy}}_{∼h,v}\right)$. We thus also estimate an instrumental variables (IV) specification in which we instrument for $s\left({\mathrm{Buy}}_{∼h,v}\right)$ with $\overset{˜}{s}\left({\mathrm{Buy}}_{∼h,v}\right)$ and ${\mathrm{Buy}}_{h,v}$ with ${\overset{˜}{\mathrm{Buy}}}_{h,v}$. The results, shown in Table 4, include point estimates that are precisely estimated and statistically indistinguishable from the OLS results. Once again, peer purchase dramatically reduces a household's installation of its own latrine. The first stage regressions show that unexpected peer purchase significantly predicts peer purchase and that unexpected own purchase significantly predicts own purchase.

**Table 4:** IV Estimates of unexpected village latrine purchase and household installation.

	First Stage	First Stage	Installation	P-value
	Own	Village	Rate	
Village unexpected purchase	−0.207	0.775		
	(−0.500, 0.020)	(0.488, 1.002)		
Unexpected purchase	0.992	−0.005		
	(0.968, 1.019)	(−0.010, −0.000)		
Village fraction HH won			−1.135	0.028
			(−3.922, −0.134)	
Household won latrine			0.146	0.001
			(0.067, 0.224)	
First Stage Wald rK F-stat	31.190			

*Notes*: Estimates reflect full sample of 1,363 households. Unexpected purchase is the difference between a household's actual latrine purchase status and its expected purchase, calculated based on the empirically observed probability that its price draw falls below its stated WTP. Village unexpected purchase is the mean of unexpected purchase across a respondent's village, leaving out the respondent's own values. Household won latrine denotes whether the household purchased a latrine through the BDM mechanism. Village fraction HH won is the mean of the Household won latrine indicators across the respondents village excluding the respondent's own value. An indicator for whether respondent was in a financing village, the respondent's WTP (in USD), and the mean village WTP (in USD) excluding the respondent are included as controls. 95% confidence intervals shown in () beneath each estimate. P-values and 95% confidence intervals are based on 100,000 Score bootstrap repetitions using Webb weights, clustering at the village level.

Finally, Table 5 replicates the OLS specification from Table 3 but includes interactions between the Financing indicator and own and peer unexpected latrine purchase. Neither of the interactions are statistically significantly different from zero. We therefore treat the estimates in Table 3, Table 4, which pool across both treatment arms, as our preferred specifications.

**Table 5:** Unexpected village latrine ownership and household installation: financing interaction.

	Installation Rate (OLS)	P-value
Village unexpected purchase	−0.674	0.204
	(−3.463, 0.448)	
Village unexpected purchase*Financing	−0.675	0.450
	(−2.523, 1.408)	
Unexpected purchase	0.156	0.004
	(0.053, 0.259)	
Unexpected purchase*Financing	−0.009	0.898
	(−0.162, 0.134)	
Observations	1363	

*Notes*: Estimates reflect full sample of 1,363 households. Unexpected purchase is the difference between a household's actual latrine purcahse status and its expected purchase, calculated based on the empirically observed probability that its price draw falls below its stated WTP. Village unexpected purchase is the mean of unexpected purchase across a respondent's village, leaving out the respondent's own values. Unexpected purchase and Village unexpected purchase are also interacted with an indicator for whether the household lived in a financing village. An indicator for whether respondent was in a financing village, the respondent's WTP (in USD) and its interaction with the financing indicator, and the mean village WTP (in USD) excluding the respondent and its interaction with the financing indicator are included as controls. 95% confidence intervals shown in () beneath each estimate. P-values and 95% confidence intervals are based on 100,000 Wild bootstrap repetitions using Webb weights, clustering at the village level.

## Discussion and conclusion

This paper shows that providing finance can dramatically increase willingness to pay for improved latrines. This finding provides evidence on the previously under-studied question of whether imperfect credit markets may be in part responsible for low WTP for environmental quality in developing countries (Greenstone and Jack, 2015). Furthermore, while micro-credit to date has not been demonstrated to provide significant benefits in terms of increasing income for clients (Banerjee, 2013, Banerjee and Karlan, 2015), this paper suggests that there may be other important ways that micro-finance can enhance welfare.^29^

This initial investment – in this context, the purchase of a latrine – is a necessary but not sufficient condition for improving environmental conditions and health. We find that, despite the large increase in WTP for the initial investment, installation rates are low even 18–24 months after purchase. An interesting question for further research is why households exhibit preferences for expensive, elaborate superstructures and what strategies or policies might encourage completing the investment in such situations.

The dramatic increase in latrine sales in financing villages has important implications for cost-effectiveness. As with many interventions, a large share of costs are village-level fixed costs, such as the time of sales agents and their transportation costs to remote villages. Since sales agents made over four times as many sales per village meeting when loans were offered, the fixed cost of their time and transportation was amortized over many more latrines sold. Of course, offering finance carries a cost, but because VisionFund was already operating in program villages, the marginal cost of managing and collecting on loans was low. Using conservative assumptions on the costs of sales and marketing, and the marginal cost of providing finance, we calculate that offering finance can reduce program costs per latrine sold by up to 70%. For example, a direct-sales intervention, in which a team of 8 full-time sales agents travel from village to village offering latrines at USD 50 without financing, would incur operational costs (sales and marketing) of USD 19 per latrine sold. Providing financing would add to operational costs by requiring a loan officer travel to process and collect loans (note we are making the conservative assumption that an additional MFI employee would be necessary, which may not be true if the MFI already has a robust presence), but even net of this financing cost, the increase in demand through financing reduces total operational costs per latrine sold to approximately 6 USD.^30^ Of course, this result is muted by the low installation rate we observed – if only roughly one in three purchased latrines are installed, then the cost to the implementer per latrine installed must be scaled up appropriately.^31^

It is important to note that the financing in our evaluation was offered only alongside the supply of materials for latrine construction and we thus cannot extend our results directly to contexts where financing is offered separately from the provision of these materials. The relevance of this limitation depends on whether one takes the perspective of a sanitation promotion organization looking to expand coverage or that of a microfinance institution looking to expand its clientele.

Finally, this paper provides a model for learning from a “decision-focused evaluation,” i.e., one designed to answer a specific programmatic question for an implementer rather than a general academic question. However, this does come at a cost, in that we are limited in our ability to explain mechanisms underlying the large effect of credit on demand. In particular, either credit constraints or impatience could explain this reduced-form result, and further research is needed to understand the relative contributions of each. Similarly, we offer evidence on the role of peer effects on installation, but credit constraints and impatience may also drive the low installation rates.

The benefit of a “decision-focused evaluation” is that implementers and policymakers can quickly adapt their programming. Financing is now much more widely available as a routine part of iDE's latrine distribution efforts, and iDE is currently working towards implementing a one-stop shop that includes installation, a shelter, and financing for both.

## Footnotes

1 25th percentile: $34.3 with financing vs. $20.0 without; median: $50.8 with financing vs. $30.0 without; 75th percentile: $76.1 with financing vs. $40.0 without. We discuss results by poverty subgroup in Section 3.3.

2 See Shah et al. (2015) for an extended discussion of the difference between decision-focused evaluations and “knowledge-focused evaluations,” those designed by researchers to address academic questions.

3 In addition to the 71% of respondents who primarily defecate in the open at baseline, we also observe individuals who report using a neighbor's latrine, a school toilet or latrine, or unimproved sanitation (for example, a hanging toilet) as their primary means of defecation.

4 In 2010, iDE received the World Toilet Organization Hall of Fame Award for its Sanitation Marketing program and resulting Easy Latrine. In 2011, VFC received the Platinum Award from MIX (Microfinance Information eXchange).

5 IDPoor 1 and 2 are the Government of Cambodia's official poverty lines: IDPoor 1 identifies “poorest or destitute” households; IDPoor 2 identifies “poor” households. IDPoor status is initially assigned based on a set of poverty indicators, then adjusted based on a consultation with village representatives to identify households with “special circumstances” (Ministry of Planning, 2008). “IDPoor” includes both IDPoor 1 and IDPoor 2.

6 Further details on logistics are provided in Section B of the Supplementary materials.

7 The Cambodia Riel is pegged to the dollar. It fluctuates within a band of 3,900 KHR/USD to 4,100 KHR/USD. We use an exchange rate of 4,000 KHR/US$ throughout this document.

8 Approximately one person-day of low-skilled labor was required for installation, which typically would cost no more than USD 5. The costs of a superstructure are often substantially higher than those of the latrine or its installation. There are three basic categories of superstructure: (a) bamboo/thatch, made from locally gathered materials; (b) tin, which can be self-constructed using materials typically costing less than $5; (c) concrete, which requires hiring a mason and costs at least USD 100. Options (a) and (b) typically require less than half a day's labor. Survey data from other villages in Kampong Thom finds an average cost of approximately USD 200, roughly 5 times the installed cost of a latrine itself. Anecdotally, households value both the durability of the concrete structure and the prestige it brings and are often reluctant to install a latrine without a fairly elaborate superstructure. This appears to have depressed installation rates in this study, as we discuss in Section 4.

9 The lowest price was down-weighted in the financing arm for budgetary reasons. Households were not informed of the distribution of prices. Although in principle the distribution of prices should not matter for an optimal bid, experimental data suggests it can e.g., Urbancic, 2011, Mazar et al., 2014). The distribution used here was chosen for simplicity.

10 In the Financing arm, the prompts and price draws for the Financing arm were in terms of the first monthly payment. The initial anchor was 30,000 KHR (USD 7.5) per month. Prices were reduced by 2,000 KHR ($0.5) per month, then increased by 1,000 KHR (USD 0.25) per month. The price draws were 8,900 KHR, 13,400 KHR, 17,800 KHR, and 22,300 KHR, which represent total payments over the year approximately equal in NPV to the price draws in the Non-financing arm. In the Non-financing arm, the lowest price received 1/20 probability weight, the second lowest received 9/20 probability weight, and the two highest recieved probability weights of 1/4.

11 See Shogren and Jason (2005) for a survey of applications of BDM in environmental economics. Horowitz (2006) points out that violations of expected utility can lead BDM bids to diverge from maximum WTP. In a study of WTP for ceramic water filters in Northern Ghana, Berry et al. (2015) find a gap of approximately USD 1 between WTP elicited through BDM and through take-it-or-leave-it (TIOLI) offers at randomized prices. For our application, we require (1) that the degree to which individuals’ preferences differ from expected utility maximization is uncorrelated with the treatment, which in expectation will hold under randomization, and (2) that the effects of non-expected utility are similar for bidding behavior under the two treatments. Under these assumptions, while we might be skeptical of the *levels* of WTP reported by participants, the *difference* in reported WTP between treatments is still informative.

12 More precisely, the household could finance the purchase through VFC. That is, the funds went directly to the latrine producer, not to the households. For convenience, we will refer to this as a “loan”, even though the household never received the principal directly. This ensured that households could not use the funds for anything other than the latrine purchase.

13 Cambodian law prohibits flat interest rates on microfinance loans, so loans were “declining balance”. That is, each month the client would pay 1/12 of the principal, plus accumulated interest on the outstanding principal. This is standard practice in VFC's local operations, and was familiar to most participants. The prices in the envelopes reflect the first month's payment, which would then decline over time.

14 Because the monthly payments fall in declining balance loans, the first month's payment served as the bid price in the Financing arm. The field team took care to explain this feature to participants to minimize the possibility that participants who were unfamiliar with declining balances misinterpret the payment amount as constant over the 12-month term and potentially under-bidding relative to their true WTP. When we convert the stream of payments to net present value for comparability with bids in the lump-sum treatment, we account for this declining payment. We view this as conservative, since it would understate the perceived NPV of a household that did not understand that payments would decline.

15 In retrospect, it likely would have been preferable to have this assessment occur before the household participated in BDM, so that the household's stated WTP would be constrained by the amount it could borrow. In our main results, we code WTP of rejected households without adjustment, and in Section 3.4 we provide robustness checks where we code rejected households’ WTP as zero or 50% of their bid.

16 Because information travels quickly in villages, it is likely that at least some of these households learned if finance was available, so while selection into not attending the meeting is random relative to treatment, there is the possibility of non-random selection into agreeing to participate the next day. However, as noted in Section 2.1, only 3 of the 1,383 households ultimately declined to participate (0.2%, two Financing, one Non-financing), so the potential for bias is low. We code these three households as having zero WTP.

17 For example, Bloom et al. (2006), Cohen and Dupas (2010), Bloom et al. (2013) and Gertler et al. (2014) obtain p-values from randomization inference, but construct confidence intervals using regression-based methods.

18 Applications in statistics and political science include Ho and Imai (2006), Small et al. (2008), Hansen and Bowers (2009). To our knowledge, the only economics papers that use randomization inference to construct confidence intervals are Barrios et al. (2012) and Quistorff (2015), both observational studies.

19 This subgroup analysis was pre-specified as Hypothesis 2 in the Pre-Analysis Plan. As pre-specified, we group both IDPoor 1 (“poorest or destitute”) and IDPoor 2 (merely “poor”) households. Given the sampling strategy described in Section 2.1, in which 30% of households were IDPoor (but not stratified by IDPoor 1 vs. IDPoor 2), our precision for estimating effects on IDPoor 1 vs. 2 is limited. The point estimates indicate that finance increased demand among IDPoor 1 households more than among IDPoor 2 households (not reported, available on request).

20 Note that in Table 1 there are significant mean differences both in the total number of cases of diarrhea and in the number of cases in children age five and younger, conditional on having such children in the household. Since less than half of the households in the sample have a child in this age range, we use just the first of these two variables as a control, but results are largely unchanged if we include a dummy for the presence of a child in the age range and code the number of such cases in households without a child in the age range a zero rather than missing.

21 The latrine parts were not stamped with identifying marks, so we cannot say with certainty that an installed latrine is the same as the one purchased in our sale. Here, we code a winning household as having installed its latrine if any improved latrine is present. In our followup survey, we collected detailed information on the type of latrine installed, if any, and the results are almost identical if we restrict the definition of “installed” to pour flush latrines with concrete rings lining the pit, consistent with the type of latrine being offered in our sale and not generally available in the area otherwise.

22 For example, Clasen et al. (2014), in an RCT studying the Total Sanitation Campaign in Orissa, India, report a program effect of +28 percentage points in the share of households with a functional latrine.

23 An alternative explanation could include present bias or procrastination: it is possible that the financing offer overcomes these biases for the purchase decision but not the installation decision. Our decision-driven evaluation limits the baseline data collected on such biases and our ability to identify them as the mechanisms for the low installation rates.

24 We supplement this descriptive analysis of screening effects with a more rigorous regression analysis in Appendix C.

25 Because a small share households in the financing arm bid an amount greater than the BDM draw price but were later not approved for a loan, Fig. 6 may slightly understate what the conditional installation rates in the financing arm would be if all households had been approved by VFC for a loan. As a robustness check, we re-estimate Fig. 6, Fig. 7 treating financing households who were rejected for the VFC loan as though they bid $0 and thus would not win the latrine at any price. The resulting figures are nearly identical to Fig. 6, Fig. 7, suggesting that the loan rejections are not meaningfully affecting our results.

26 This aspiration for very high-end, expensive facilities resembles that described in Northern India by Coffey et al. (2014), who report that the minimally acceptable latrine described by their respondents would cost, on average, $350 to construct.

27 There is substantial evidence of latrine sharing across households at both baseline and endline. At baseline, over 12% of households have at least one member who primarily uses a neighbor's latrine for defecation. Note that this method understates the true likelihood of latrine sharing – any household with a member who uses a neighbor's latrine occasionally but not as their primary defecation site, will not be counted. At endline, households with an installed latrine are asked whether their latrine is co-owned with another household and whether any other households regularly use the latrine. Over 21% of households with a latrine installed report either co-owning the latrine with another household or sharing their latrine regularly with other households.

28 The draws in the Financing treatment were in terms of monthly payments corresponding to total payments with net present value equal to $20, $30, $40, $50.

29 Other examples include micro-finance (loans or savings) increasing demand for health goods (Devoto et al., 2012, Alessandro Tarozzi et al., 2014, Guiteras et al., 2015a) and micro-savings increasing business growth (Dupas and Robinson, 2013).

30 Detailed assumptions and calculation available on request.

31 For example, applying the 1/3 installation rate to the inverse demand curves observed in our sample, selling at USD 50 with financing would lead to approximately a 17 pp reduction in the share of households without an installed latrine, vs. a reduction of approximately 4 pp from sales without financing at the same price.

Appendix A Supplementary data associated with this article can be found in the online version at doi:10.1016/j.jeem.2016.11.004.
